# *Peroxicretion*: a novel secretion pathway in the eukaryotic cell

**DOI:** 10.1186/1472-6750-9-48

**Published:** 2009-05-20

**Authors:** Cees MJ Sagt, Peter J ten Haaft, Ingeborg M Minneboo, Miranda P Hartog, Robbert A Damveld, Jan Metske van der Laan, Michiel Akeroyd, Thibaut J Wenzel, Francisca A Luesken, Marten Veenhuis, Ida van der Klei, Johannes H de Winde

**Affiliations:** 1 DSM Biotechnology Center, Beijerinck Laboratory, PO Box 1, 2600MA Delft, the Netherlands; 2 Department of Microbiology Radboud University Nijmegen Toernooiveld 1, 6525ED Nijmegen, the Netherlands; 3 Groningen University department of microbiology, Groningen, the Netherlands; 4 Kluyver Centre for Genomics of Industrial Fermentation, Delft University of Technology, Department for Biotechnology, Julianalaan 67, 2628BC Delft, the Netherlands

## Abstract

**Background:**

Enzyme production in microbial cells has been limited to secreted enzymes or intracellular enzymes followed by expensive down stream processing. Extracellular enzymes consists mainly of hydrolases while intracellular enzymes exhibit a much broader diversity. If these intracellular enzymes could be secreted by the cell the potential of industrial applications of enzymes would be enlarged. Therefore a novel secretion pathway for intracellular proteins was developed, using peroxisomes as secretion vesicles.

**Results:**

Peroxisomes were decorated with a Golgi derived v-SNARE using a peroxisomal membrane protein as an anchor. This allowed the peroxisomes to fuse with the plasma membrane. Intracellular proteins were transported into the peroxisomes by adding a peroxisomal import signal (SKL tag). The proteins which were imported in the peroxisomes, were released into the extra-cellular space through this artificial secretion pathway which was designated peroxicretion. This concept was supported by electron microscopy studies.

**Conclusion:**

Our results demonstrate that it is possible to reroute the intracellular trafficking of vesicles by changing the localisation of SNARE molecules, this approach can be used in *in vivo *biological studies to clarify the different control mechanisms regulating intracellular membrane trafficking. In addition we demonstrate peroxicretion of a diverse set of intracellular proteins. Therefore, we anticipate that the concept of peroxicretion may revolutionize the production of intracellular proteins from fungi and other microbial cells, as well as from mammalian cells.

## Background

The specificity of intracellular membrane trafficking is determined by multiple layers of control mechanisms that ensure that only appropriate organelles fuse with specific target compartments. These include Rab-GTPases [1] operating in conjunction with polyphosphoinositides [2] and Rab effectors [3] that frequently include multiprotein complexes. In eukaryotes, membrane fusion of secretory vesicles is mediated by SNARE-proteins [4] and specificity of membrane fusion is obtained by specific SNARE-protein interactions. In yeast, fusion of post-Golgi trafficking vesicles requires at least 10 genes including the Rab-GTPase Sec4 [5] the Exocyst multiprotein complex [6] and the SNAREs Snc1/2 [7] on the transport vesicle and Sso1/2 [8] and Sec9 [9] on the plasma membrane. Moreover organelles can only fuse with target membranes once they are transported into close proximity, involving directed transport along cytoskeletal tracts [10]. The formation of the resulting SNARE-pin subsequently triggers membrane fusion [11]. The ER supplies the secretory route with membrane enclosed vesicles which travel from the ER via the Golgi towards the cell membrane.

The ER is very different from the cytosol regarding post-translational protein modifications. *N*-glycosylation of proteins in the ER is important for folding, degradation and quality control [12]. The cytosol does not contain an *N*-glycosylation machinery and as a consequence, soluble cytosolic proteins are not *N*-glycosylated [13]. In addition, the reducing environment of the cytosol is very different from that in the ER and Golgi, where oxidizing conditions and specialized folding enzymes like Pdi1 and Ero1 facilitate disulfide bridge formation [14]. These fundamental differences between secretory pathway and cytosol complicate the routing of cytosolic proteins through the secretory pathway to yield active, secreted enzymes. In fact, literature does not describe any successful extracellular production of an intracellular protein through the secretory pathway. Cytosolic proteins preferentially fold into their active conformation with the aid of specific chaperones and folding enzymes, under the reducing conditions which are normal to the cytosol [15]. Recently it has been described that peroxisomes also may have their origin in the ER [16]. However they do not fuse with other compartments and SNARE molecules have not been detected on peroxisomes [17]. The peroxisome has all the necessary features to enable import of completely folded and mature intracellular proteins [18]. Proteins of the peroxisomal lumen contain either a PTS1 [19] or a PTS2 signal [20]. The PTS1 signal is a specific tripeptide located at the C-terminal end of the protein, and is recognized by the Pex5 receptor, a translocator for PTS1 containing proteins [21]. The ER origin of peroxisomes, combined with their capacity to import completely folded proteins, would render them ideally suited for secretion of intracellular proteins.

**Figure 1:**
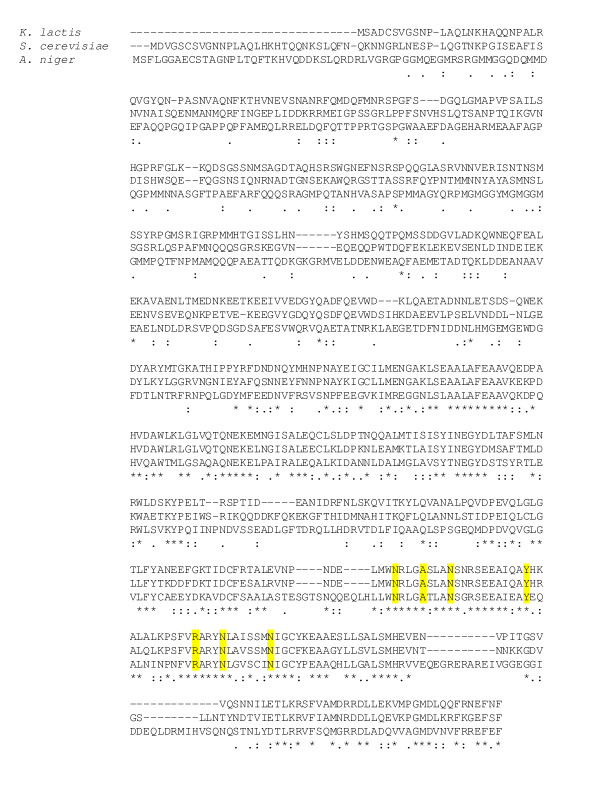
**Alignment of the PEX5 orthologs of *S. cerevisiae*, *A. niger *and *K. lactis***. Identical aminoacids are indicated with *, highly similar aminoacids are indicated with :, similar aminoacids are indicated with •. The conserved aminoacids which are important for PTS1 recognition are colored in yellow.

To enable this we have decorated *Aspergillus niger *peroxisomes with the *A. niger *ortholog of the v-SNARE Snc1 (SncA), by expressing it as a chimera with the *A. niger *ortholog of the peroxisomal membrane protein Pmp22 (PmpA) [22]. In Figure 1 panel C a schematic representation of the fusion of peroxisomes with the plasmamebrane is shown. The modified peroxisomes were able to fuse with the plasma membrane as evidenced by electron microscopy and extracellular secretion of peroxisomal accumulated proteins, which were tagged with the PTS1-signal peptide -SKL. We have named this novel technology peroxicretion, for peroxisome-mediated intracellular protein secretion.

## Results

### PTS 1 mediated peroxisomal import in *A. niger*

**Figure 2:**
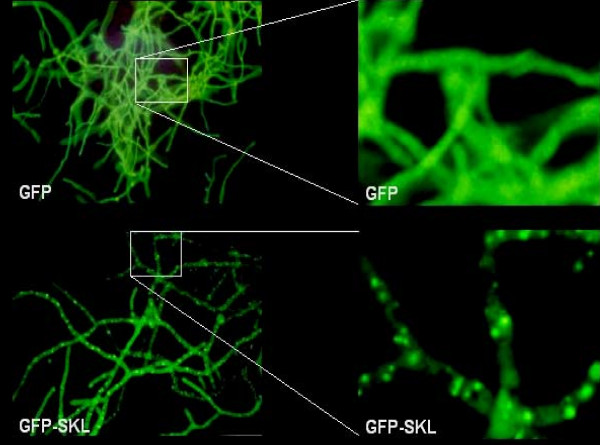
**Peroxisomal localisation of eGFP-SKL**. Fluorescence microscopy was applied to determine the sub cellular localisation of eGFP-SKL. As shown in the right panel the SKL tagged eGFP is found in a punctuated pattern whereas eGFP without SKL is localised throughout the cytosol as shown in the left part of the panel.

The PTS1 receptor Pex5 is responsible for recognition and transport of PTS1-containing proteins into peroxisomes [21]. To confirm that PTS1 signals will result in peroxisomal localization in *A. niger *we have identified a Pex5 ortholog in the genome of *A. niger *(Genbank 4989140). Amino acids important for PTS1 recognition are conserved in the Pex5 ortholog (figure 1), suggesting that the presence of an -SKL sequence at the C-terminus of model proteins will lead to peroxisomal localization. Indeed, expressing -SKL tagged eGFP in *A. niger *(figure 2) caused a punctated pattern typical for peroxisomal localization. This result indicated that -SKL mediated peroxisomal targeting occurs in *A. niger *as expected based on the presence of a Pex5 receptor ortholog.

### Decoration of *A. niger *peroxisomes with v-SNARE molecules

**Figure 3:**
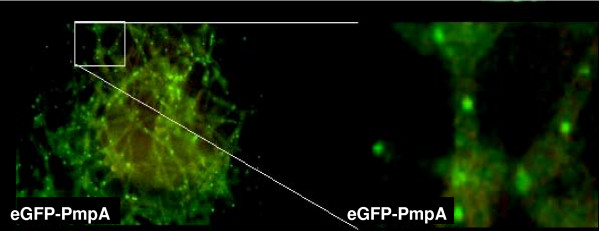
**Peroxisomal localisation of eGFP-PmpA**. The eGFP tagged PmpA showed a similar localisation pattern compared to eGFP-SKL (figure 2), indicating that eGFP-PmpA also localises to peroxisomes.

**Figure 4:**
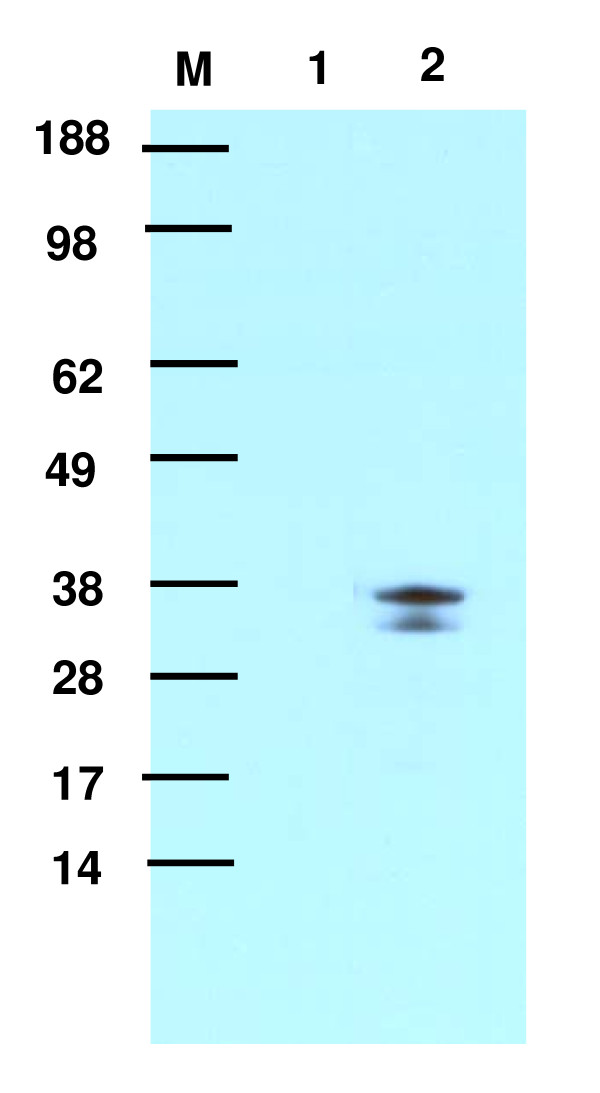
**Expression of the fusion peptide SncA-PmpA**. Cell free extracts were obtained from untransformed *A. niger *(lane 1) and from SncA-PmpA transformed *A. niger *(lane 2). The Cell free extracts were subjected to SDS-PAGE and western blotting according the manufacturers instructions (Invitrogen) and detection was performed using a custom made antibody against SncA protein (Eurogentec). The expected size of the SncA-PmpA fusion protein is 35 kDa.

**Figure 5:**
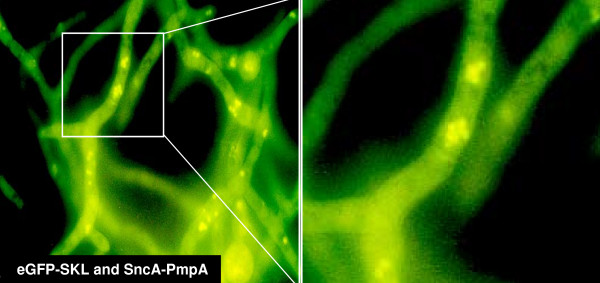
**Co-expression of eGFP-SKL and SncA-PmpA in *A. niger***. *A. niger *transformants were grown on MEAM as described in materials and methods. After 48 hours biomass was transferred to glass slides and subjected to fluorescence microscopy. The decoration of peroxisomes with the v-SNARE SncA results in clustering of peroxisomes.

To enable fusion of peroxisomes with the plasma membrane it was necessary to identify a peroxisomal membrane anchor, which could be used to place the v-SNARE SncA on the peroxisome. This peroxisomal membrane anchor should have the N-terminus positioned towards the cytosol, enabling N-terminal fusions. The resulting chimeric protein is anchored in the peroxiomal membrane with the N-terminal fused SncA positioned at the cytosolic side of the peroxisome. Using the CBS prediction server  we predicted the topology of the *A. niger *ortholog of Pmp22, which has been studied in *Arabidopsis *and in mammalian cells [22,23]. A membrane topology was predicted of 4 TMD's with the N-terminus positioned at the cytosolic side of the peroxisome. PmpA contains two peroxisomal targeting regions with similar clusters of basic amino acids, interacting with Pex19p [22]. This prediction is in agreement with experimental evidence determining the topology of Pmp22 in *Arabidopsis *and mammalian cells [22,23]. To determine whether the Pmp22p ortholog of *A. niger *localises to the peroxisomes we constructed an *eGFP-pmpA *chimera and expressed this fusion gene in *A. niger*. We determined the localization of the eGFP-PmpA fusion protein by fluorescence microscopy. A similar punctated pattern was observed as with the eGFP-SKL fusion construct (figure 3), indicating that PmpA indeed can be used as peroxisomal membrane anchor. Moreover the majority of the eGFP-PmpA fusion protein is localised in peroxisomes. Since the SncA-PmpA fusion construct expressed using the same GlaA promoter in identical expression cassettes, the majority of SncA-PmpA will be localised to peroxisomes as well, this is shown in figure 4). The v-SNARE Snc1 in yeast, is intimately involved in fusion of Golgi derived vesicles to the plasma membrane. We have identified an Snc1 ortholog in *A. niger *(*sncA*), and fused the gene (without its transmembrane coding region) to *pmpA*. When GFP-SKL and SncA-PmpA are co-overexpressed, a clustering of peroxisomes is observed (figure 5). This clustering is not observed when GFP-SKL is overexpressed (figure 2). This indicates that the SncA part exposed to the cytosol is interacting with itself thereby causing perxosimes to cluster. This behaviour of v-SNAREs has been observed before [24] and could be enhanced when overexpressed.

**Figure 6:**
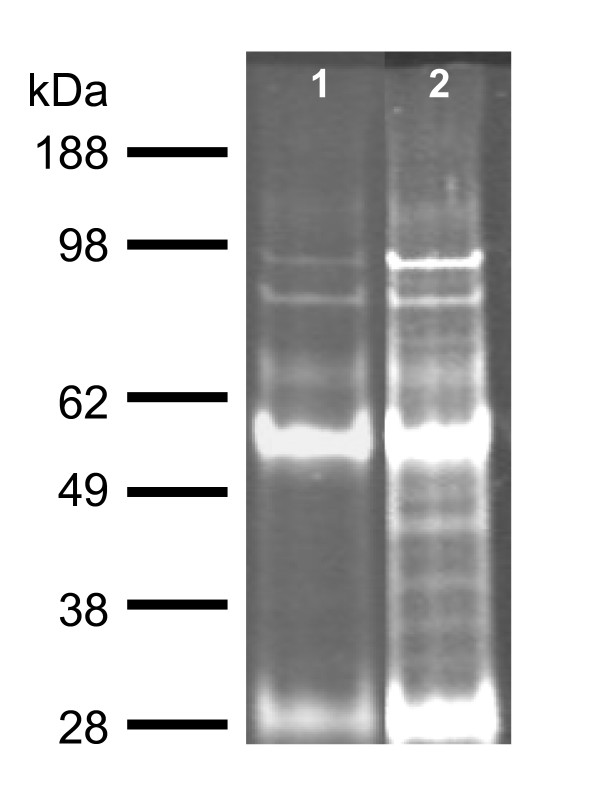
**Analysis of co-immunoprecipitation complexes using anti-SncA by SDS-PAGE and Sypro ruby**. SDS-PAGE (4–12% Bis-Tris) gel loaded with the immunoprecipitation samples of wild type strain (lane 1) and peroxicretion strain (wild type strain transformed with SncA-PmpA fusion construct) (lane 2) was stained with Sypro Ruby. The heavy and light chain of the used antibodies are running at 55 kDa and 28 kDa. Proteins Differential bands were identified by MS/MS.

Using co-immunoprecipitaion we were able to identify the *A. niger *homologue of fox2p as a partner of the SncA-PmpA fusion protein (figure 6). Fox2p is a peroxisomal protein involved in beta oxidation [25]. This is in line with the data presented which indicates the peroxisomal localisation of SNCA/PMPA.

### Peroxicretion in *A. niger*

**Figure 7:**
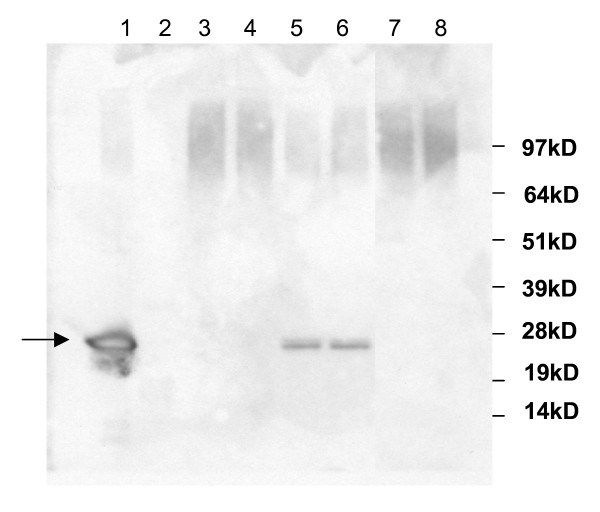
**Secretion of eGFP-SKL mediated by SncA decorated peroxisomes**. *A. niger *strains were cultivated for 24 hours in MEAM. 10 ul samples of the medium were taken and subjected to SDS-PAGE and subsequently to western blotting as indicated by the manufacturer (Invitrogen). Lane 1: positive control eGFP, lane 2: MW marker indicated at the right hand side, lane 3 and 4: eGFP-SKL transformants, lane 5 and 6: eGFP-SKL, SNCA-PMPA co-transformants, lane 7 and 8: untransformed wild type strain.

**Figure 8:**
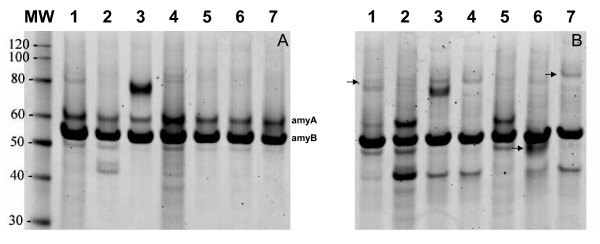
**Peroxicretion of putative peroxisomal proteins**. SDS-PAGE analysis of shake-flask samples of the control strains (panel A) and peroxicreting strains (panel B), peroxicreting different intracellular *A. niger *enzymes as indicated. When indicated an SKL sequence was added to the C terminus of the protein. After SDS-PAGE the cells were stained with coomassie brilliant blue. Endogenous amylases are indicated. The strains were grown for four days at 30°C, 250 rpm In MEAM. Supernatant was isolated and loaded on a Nupage gel. Lane 1: strong similarity to catalase/peroxidase CpeB, 84 kDa (An01g01830), lane 2: strong similarity to chitinase 1 precursor Cts1, 48 kDa (An02g07020) an SKL sequence was added to the C terminus of the protein, lane 3: strong similarity to alpha-amylase precursor AmyA, 60 kDa (An09g03100) an SKL sequence was added to the C terminus of the protein, lane 4: strong similarity to lipase LipP, 37 kDa (An09g06390) an SKL sequence was added to the C terminus of the protein, lane 5: similarity to endo-1,4-beta-xylanase XynD, 35 kDa (An11g03120) an SKL sequence was added to the C terminus of the protein, lane 6: strong similarity to D-amino acid oxidase Dao1, 41 kDa (An14g05380) an SKL sequence was added to the C terminus of the protein, lane 7: show strong similarity to several fungal alcohol oxidases, 74 kDa (An18g05480).

**Figure 9:**
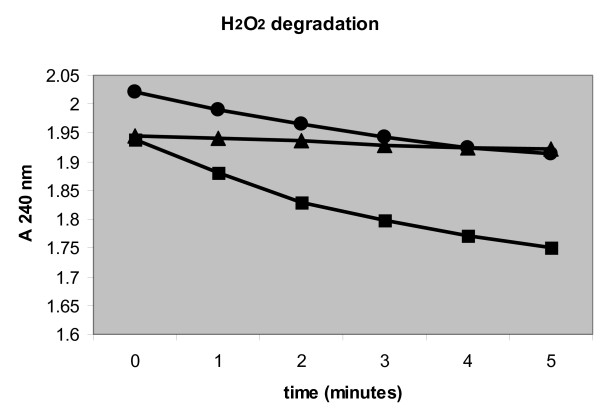
**Enzymatic activity of peroxicreted alcohol oxidase**. Using 100 μl of supernatant of shakeflask grown *A. niger *cultures the degradation of 0.1% H_2_O_2 _was monitored in 2 ml milliQ by measuring absorption at 240 nm. (black triangle) represents the H_2_O_2 _degrading activity of 100 μl supernatant of the wild type strain, (black circle) represents the H_2_O_2 _degrading activity of 100 μl supernatant of the peroxicretion strain and (black square) represents the H_2_O_2 _degrading activity of 100 μl supernatant of the peroxicretion strain overexpressing gene ID 4990113 which shows strong similarity with several alcohol oxidases.

We cultivated *A. niger *co-expressing an SncA-PmpA fusion protein and an SKL-tagged eGFP in MEAM cultures as described in the materials and methods. After 24 hours, only transformants containing SncA-PmpA secreted eGFP-SKL into the extracellular medium, as evidenced by Western blot analysis using anti GFP antibody (figure 7). Expression of *sncA-pmpA *does not result in enhanced levels of extracellular acetamidase activity (used as selection marker), indicating that cell lysis is only limited. However the peroxicretion efficiency was also determined and in the peroxicretion strain, overexpressing SncA-PmpA and eGFP-SKL, 55% of the total GFP was extracellular. When we expressed only GFP-SKL we determined 25% of the total GFP in the supernatant. This indicates that less than 50% of the extracellular GFP is due to lysis and more than 50% due to actual peroxicretion. We conclude that decoration of peroxisomes with the v-SNARE SncA resulted in fusion of peroxisomes with the plasma membrane, causing release of peroxisomal content in the extracellular medium. The applicability of this approach to secrete intracellular enzymes was further investigated using a set of enzymes indicated in figure 8. We have expressed the indicated proteins in wild type *A. niger *(panel A) and in a peroxicreting *A. niger *(panel B), when indicated an SKL tag was placed at the C terminus of the indicated proteins. The amylase proteins (amyA and amyB) are also visible, the presence of amyA is pH dependent. This explains why amyA is not always visible. The peroxicretion strain shows a slightly different acidification profile compared to the wild type strain. Results clearly showed peroxicretion of 3 overexpressed putative peroxisomal proteins from a SncA-PmpA expressing strain (figure 8). Using MS/MS we could corroborate peroxicretion of at least one of those 3 proteins (strong similarity to catalase/peroxidase CpeB, (An01g01830)) and in addition identified one extra peroxicreted protein (similarity to endo-1,4-beta-xylanase XynD, (An11g03120)). The relatively low abundance of these proteins is likely to be caused by exposure to oxidised conditions combined with the presence of extracellular proteases. It is evident that putative peroxisomal proteins can be peroxicreted as well as cytosolic proteins like the XynD orthologue. However the peroxisomal enzymes have a higher success rate probably because they are adapted to peroxisomal conditions in contrast to cytosolic proteins. Simple C-terminal SKL addition is sufficient to peroxicrete the XynD orthologue in SncA-PmpA expressing cells. Proteins which contain a putative PTS1 sequence like the catalase/peroxidase CpeB orthologue (An01g01830) and the alcohol oxidase orthologue (An18g05480) could be peroxicreted without modifications. The peroxicreted alcohol oxidase shows enzymatic activity in an H_2_O_2 _degrading assay, described in [26], and depicted in figure 9. The wild type strain shows almost no H_2_O_2 _degrading activity in the supernatant whereas the peroxicretion strain shows an increasing in H_2_O_2 _degrading activity in the supernatant. This is most likely due to peroxicretion of endogenous catalases/peroxidases localised in peroxisomes.

### Finetuning of peroxicretion

**Figure 10:**
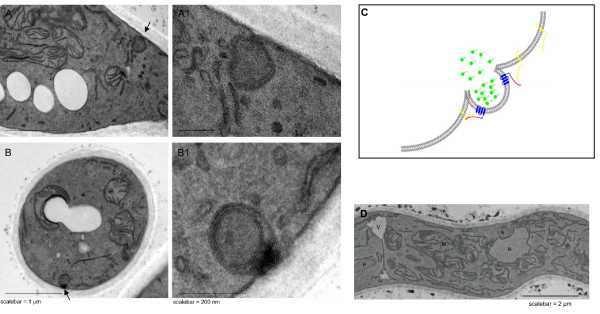
**Survey of hyphal cells showing the position of a peroxisome (arrow) in the vicinity of the cell membrane in SncA-PmpA expressing *A. niger***. Panel A. Lengthwise overview of cellular membranes in SncA-PmpA expressing *A. niger*. Arrow indicates continuity of peroxisomes with the cell membrane (high magnification in panel A1). Panel B. Crosswise overview of cellular membranes in SncA-PmpA expressing *A. niger*. Arrow indicates continuity of peroxisomes with the cell membrane (high magnification in panel B1). Panel C. Schematic representation of the release of the peroxisomal content due to fusion of the peroxisome with the plasmamembrane. The target SNARE (Sso1 ortholog) is shown in yellow as a transmembrane protein. The chimeric protein SncA-PmpA is depicted in blue (PmpA part) and red (SncA part). For simplicity Sec9 ortholog is not shown. The release of peroxisomal content is depicted, demonstrating peroxicretion of SKL tagged proteins (in green). Panel D. Detail of a glucose-grown *A. niger *WT cell, showing the presence of peroxisomes that are randomly scattered in the cytosol. M-mitochondrion, N – nucleus, P – peroxisomes, V-vacuole.

**Figure 11:**
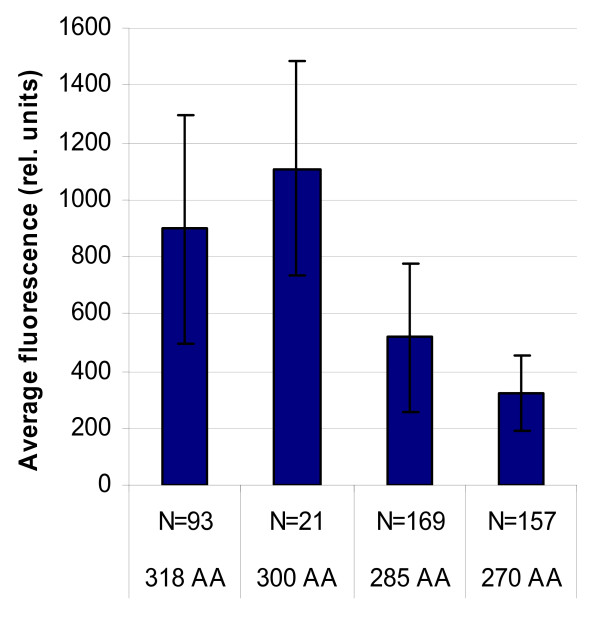
**Mean fluorescence of eGFP-SKL overexpressing strains containing different truncated SncA-PmpA constructs**. Fluorescence of individual samples was measured in supernatant of MTP cultures. SD is indicated as well as the number of transformants which were analysed.

Fusion of peroxisomes with the plasma membrane was supported using electron microscopy. Inspection of ultrathin section of KMnO_4_-fixed cells revealed that peroxisomes were frequently located in close vicinity of the cell membrane and often showed continuation with this membrane (figure 10A, B, C.). This was never observed in wild type cells without SncA-PmpA expression in which the organelles are scattered throughout the cytosol but are not seen in close proximity of the cell membrane (figure 10D). The efficiency of peroxicretion is likely to be controlled at the level of SNARE-pin formation during membrane fusion. In order to increase this efficiency of SNARE pin formation we have truncated the cytoplasmic tail of PmpA in order to place the v-SNARE SncA in closer proximity to the peroxisomal membrane. The peroxicretion efficiency is reduced when the N-terminus of PmpA is truncated with more than 18 amino acids, probably due to mislocalization (figure 11).

**Figure 12:**
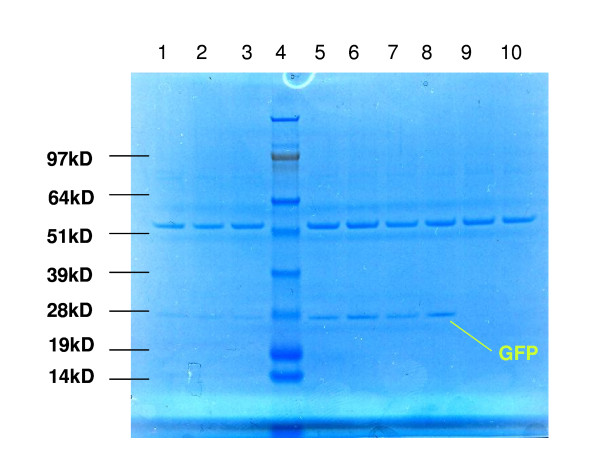
**Ceramide stimulates peroxicretion, overexpression of PEX11 ortholog (An11g02590) does not**. 10 μl of supernatant after 48 hours growth of the strains transformed with the indicated constructs at 30°C, 250 rpm in MEAM. Lane 1; eGFP-SKL, lane 2; eGFP-SKL and C2 ceramide, lane 3; eGFP-SKL (duplo of lane 1), lane 4; MW, lane 5; eGFP-SKL and SncA-PmpA, lane 6; eGFP-SKL, C2 ceramide and SNCA-PMPA, lane 7; eGFP-SKL, SncA-PmpA and PEX11 ortholog, lane 8; eGFP-SKL, C2 ceramide, SncA-PmpA and PEX11 ortholog lane 9; WT, lane 10; WT and C2 ceramide.

**Figure 13:**
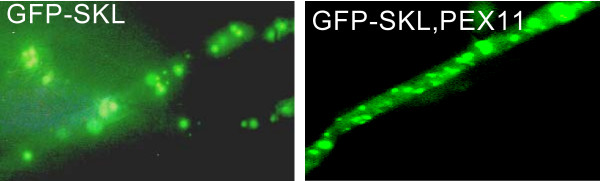
**Overexpression of PEX11 ortholog leads to increased peroxisomal proliferation in *A. niger***. PEX11 ortholog overexpression leads to a minor increase in the number of peroxisomes however the peroxisomes seems to be smaller compared to the strain where only eGFP-SKL was expressed.

Another way of increasing the peroxicretion efficiency is to use C2 ceramide. Activation of CAPP by adding C2-ceramide is known to result in increased availability of t-SNARE, Sso1p, which is important for SNARE-pin formation [26,27]. Indeed, addition of C2-ceramide slightly enhanced the peroxicretion efficiency (figure 12). A third approach to increase the efficiency of peroxicreion is to increase the number of peroxisomes. Overexpression of the *A. niger *ortholog of Pex11, which is known to be involved in peroxisomal proliferation [21] only resulted in minor peroxisomal proliferation in the *A. niger *transformants, while peroxicretion was not enhanced (figure 13). This may be explained by the fact that the increase in organelle numbers is not associated with a concomitant increase in matrix protein levels.

## Discussion

In this study the v-SNARE SncA without its transmembrane domain was placed on the peroxisome using PmpA as a membrane anchor. The transmembrane domain of Snc1p is reported to be important for its function [28]. However, replacing the SncA-TMD by PmpA did not diminish the potential of SncA to enforce membrane fusion, since the peroxisomal content was released in the extracellular environment. PmpA as membrane anchor is sufficient for SncA to bring the membrane bilayers in close contact so that spontaneous membrane fusion occurs. We have selected peroxisomes because they can import completely folded proteins, which makes them ideally suited to transport and secrete proteins which are normally are localized intracellular. Recently, convincing evidence was presented that peroxisomes may originate from the ER [16]. This is important since the lipid composition of peroxisomes now is likely to be comparable to that of other ER/Golgi derived organelles like other secretory vesicles. Because of this similarity in lipid composition it appears unlikely that lipid incompatibility between peroxisomes and the plasma membrane would present a major hurdle for peroxicretion. Moreover, the small GTPase Rho1 is localized on peroxisomes through interaction with the peroxisomal membrane protein Pex25 [29]. Rho1p is known to play a role in actin reorganization and membrane dynamics. In yeast, Rho1 regulates polarized growth, and in the filamentous fungus *A. niger *polarized growth is even more predominant. In *Arabidopsis*, transportation of peroxisomes by actin filaments is reported [30] similar to transportation of secretory vesicles. This would be a possible mechanism how peroxisomes are able to be transported intracellular, Snc1 decorated peroxisomes are able to fuse with the plasmamembrane. The examples of Peroxicretion as described in this paper, shows that positioning of SncA on the peroxisomal membrane is sufficient for fusion of the peroxisome with the plasma membrane and subsequent release of its cargo. Interestingly, all these findings strengthen the notion that peroxisomes are derivatives of the secretory pathway. This renders peroxisomes as attractive vehicles for the transport of intracellular proteins towards the plasma membrane and secretion into the extracellular medium. It is however not excluded that peroxismes fuse to other intracellular compartments like endosomes before actual fusion with the plasmamebrane takes place. It is very likely that not all peroxisomes will be fused to the cellmembrane since the localization of PmpA fused proteins is not absolutely peroxisomal. In addition we do not observe decreased growth under conditions were peroxisomes are expected to be important. It is more likely that the peroxicretion concept as presented in this paper is not yet very efficient and that several key steps in the process like fusion of peroxisomes with the cell membrane, peroxisomal import of proteins to be peroxicreted and stability of these proteins have to be improved.

## Conclusion

This paper shows that it is possible to redirect intracellular trafficking of organelles by re-localizing v-SNARE molecules. The decoration of target organelles with selected v-SNARE proteins is facilitated by using a modified membrane anchor which positions the v-SNARE onto the membrane of the target vesicle. This technology opens up numerous possibilities for studying intracellular vesicle trafficking *in vivo*. Besides this fundamental application the redirection of intracellular organelles also can be used in industrial biotechnology. As demonstrated in this paper, the targeting of proteins towards peroxisomes by using a C-terminal SKL sequence followed by fusion of modified peroxisomes with the plasmamembrane results in secretion of intracellular proteins. This process is designated peroxicretion. Although the levels of the peroxicreted products are still very low it shows the potential of the peroxicretion technology.

## Methods

### Constructs, strains and transformation

Standard molecular cloning techniques were performed. The constructs pGBFINSNP-2 to 5 contain the v-SNARE named *sncA *(An12g07570), fused to the N-terminal 318, 300, 285, or 275 amino acids of the peroxisomal membrane protein *pmpA *(An04g09130) respectively. The *sncA-pmpA *fusion gene is deposited as: GenBank DQ768214. Both genes were obtained by PCR on genomic DNA and the obtained nucleotide sequence was confirmed by sequencing (Baseclear, Leiden). The vector pGBFINGFS-1 contains eGFP with the amino acids SKL added to the C-terminus. The nucleotide sequence (5'-TCCAAGCTC-3') encoding for the amino acids SKL was introduced at the C-terminus of eGFP by PCR. The construct pGBFINGFM-2 (GenBank DQ768213) was obtained by translational fusion of the eGFP and *pmpA *ORF through PCR. The pGBFIN vector was also used for overexpression of different genes (An01g01830, An02g07020, An09g03100, An09g06390, An11g03120, An14g05380 and An18g05480). Using primers which were extended by 9 nucleotides encoding SKL the protein encoding sequences of An02g07020, An09g03100, An09g06390, An11g03120 and An14g05380 were modified at the C terminus. All of the above *A. niger *expression constructs are driven by the strong glucoamylase promoter and harbour flanking regions to ensure convenient targeting and expression in the fungal host as described previously [31]. Subsequent transformation of *A. niger *strain CBS 513.88 with the expression constructs was performed as previously described [32].

### Culture methods

*A. niger *strains were inoculated at 5 × 10^6 ^spores/ml MEAM consisting of: 6 g NaNO_3_; 0.52 g KCl; 1.52 g KH_2_PO_4_; 1.12 ml 4 M KOH; 0.52 g MgSO_4 _^.^7H_2_O; 10 g glucose; 1 g casaminoacids; 22 mg g ZnSO_4 _^.^7H_2_O; 11 mg H_3_BO_3_; 5 mg FeSO_4 _^.^7H_2_O; 1.7 mg CoCl_2_. 6H_2_O; 1.6 mg CuSO_4_. 5H_2_O; 1.5 mg Na_2_MoO_4_. 2H_2_O; 50 mg EDTA; 5 mg MnCl_2_. 2H_2_O; 2 mg riboflavin; 2 mg thiamin-HCl; 2 mg nicotinamid; 1 mg pyrodoxin-HCl; 0.2 mg pantothenic acid; 4 μg biotin;10 ml penicillin/streptomycine (Invitrogen) per liter. The strains were grown at 30°C and 250 rpm for 1–3 days. When required conidiospores were obtained by growth on Potato Dextrose Agar (PDA, Oxoid, England) for 5 days at 30°C, and isolated with MilliQ and a spatula.

### Western blot analysis

SDS-PAGE was performed using NuPAGE Novex Bis-Tris precast gels (Invitrogen) according to the supplier's manual. Proteins were visualized by staining with SimplyBlue SafeStain (Invitrogen). Western analysis was performed with the XCell II semi-wet blotting module (Invitrogen) using MOPS buffer and nitrocellulose membrane (0.45 μm pore size) according to the supplier's manual. GFP was specifically detected by using 1:1,000 fold diluted GFP monoclonal antibody (Covance, California). After incubation with secondary antibody conjugated to horseradish peroxidase (anti-mouse, 1:1,000 dilution, PIERCE), immunoreactive proteins were detected by the enhanced chemiluminescence system (ECL, Amersham Pharmacia) and exposed to radiographic film (Kodak). The SNC 1 westerns were prepared similar to the procedure described above with the expection that the anti-SNC1 antibody was custom made by Eurogentec.

### Immunoprecipitation of SncA-pmpA

Frozen cells were disrupted in a mortar filled with liquid nitrogen and suspended in 0.5 ml 20 mM Sodium-Phosphate, 1% TritonX-100, 1 mM EDTA, and protease inhibiter. Cell lysate was voraciously vortexed and placed on ice for 10 minutes. TritonX-100 induces lyses of *A. niger*. The cell lysate was centrifuged for 5 minutes at 13.000 g and supernatant was taken. A pre-clearance step with 25 μl was used to reduce the background of a-specific bonding to Protein-A-Sepharose (10% Protein-A-Sepharose in 20 mM Sodium phosphate pH 7,4, 1 mM EDTA, 0,1% Triton X-100). The soluble fraction was incubated with Protein-A-Sepharose and rotated head over head for 1 hour at 4°C. The supernatant was incubated with 2.5 μl 10^-5 ^diluted antibody SNC α-SNC (serum 2^e ^booster NL 03077, rabbit no = SN1391) for 1 hour at 4°C to bind the SNC for 1 hour at 4°C. In total 25 μl Protein-A-Sepharose was added to the sample to bind antibody SNC with bounded SNC and the incubation was extended for again one hour. Protein-A-Sepharose was used to facilitate spin-down antibody SNC with bounded SNC. To remove the remaining antibody, Protein-A-Sepharose and the not bound proteins, the sample was washed once for 5 minutes with 1,0 ml 20 mM SodiumPhosphate, 0,1% TritonX-100, 1 mM EDTA, and protease inhibiter. The samples were treated with 25 μl sample buffer and 5 μl reducing agent. After heating the samples for 5 minutes at 95°C and centrifuging, 20 μl supernatant was loaded on the 4%–12% SDS-PAGE gel. After electrophoresis, Sypro Ruby staining was performed. For 24 hours the gel was stained in 100 ml Sypro Ruby staining. After staining, the gel was washed once with MilliQ. Instead of a Coomassie blue staining the gel was stained with a Sypro Ruby, because this is more sensitive. Sypro ruby does not need to be destained, because the dye does not bind irreversible to proteins and is therefore washable from the sample (Patton, 2000). The fragments were cut out of the gel and identified by mass-spectrometry.

### Microscopy

For analysis of eGFP localization the fungal cells were grown at 30°C in MEAM for 1–2 days. Mycelium was transferred to microscope coverslides and studied under a Leica DMLA microscope connected to a CTRMIC unit. The apparatus was controlled by Qwin software from Leica. Electron microscopy was performed as described before [33].

### Digestion and LC-MS/MS analysis

The sups were filtered over centrifugal devices (Pall) in tubes. Proteins with MW >100 kDa were filtered over 100 kDa centrifugal devices, proteins with MW 30–100 kDa were filtered over 30 kDa centrifugal devices and proteins with MW 10–30 kDa were filtered over 10 kDa centrifugal devices. 500 μL MQ was added on the filters and again the samples were centrifuged at 13000 rpm 4°C for 15 minutes. 150 μL 80 mM NH_4_HCO_3 _was added to the retentate of each of the samples after filtration and the retentate was transferred to 1.5 mL eppendorf tubes after pipetting up and down on the filter a couple of times. The proteins were denatured by incubation at 97°C for 10 minutes. 350 μL 80 mM NH_4_HCO_3 _and 20 μL 250 μg/mL trypsin were added and the proteins were digested by incubation at 37°C over night. 6 μL 100 mM DTT was added and the samples were incubated at room temperature for 30 minutes. LC-MS/MS was performed on the CapLC-QTOFII (Waters) system. For each of the samples a different MS/MS method was made with the selected precursors for the over-expressed proteins. For each of the precursors theoretical fragmentation (MS/MS) spectra were made using Masslynx software (Waters) and the LC-MS/MS data was compared to these theoretical fragmentation spectra.

### Quantification of fluorescence

Approximately 1 × 10^5 ^conidiospores were inoculated in 300 μl MEAM (vitamins were omitted because of interference with the fluorescence measurements) per MTP well. After 5 days incubation at 30°C in an MTP (Nunc) the medium was separated from the mycelium and 200 μl was transferred to a new MTP (Greiner, Fluotrac 200). Subsequently the fluorescence was measured on a Gemini spectra MAX (Molecular devices) controlled by SOFT max PRO v3.1.1 (Molecular Devices) using an excitation wavelength of 490 nm and an emission wavelength of 510 nm. Additional settings: cut-off 495 nm, PMT auto, calibrate on, 6 reads per well, 9 points per well. The amount of eGFP-SKL was determined in RFUs (relative fluorescent units).

## Abbreviations

SNARE: Soluble Nsf-Attachment protein Receptors; ER: Endoplasmic Reticulum; PTS: peroxisomal Targeting Sequence; eGFP: enhanced Green Fluorescent Protein; TMD: Trans Membrane Domain; CAPP: Ceramide Activated Protein Phosphatase; MEAM: Minimal Enriched Aspergillus Medium.

## Authors' contributions

The experiments were conceived and designed by CS, TW, RD and PtH. MH and JdW performed the PmpA truncation studies and PtH performed the peroxicretion experiments. IM constructed the *sncA-pmpA *fusion gene which was used throughout this study. FL performed the peroxicretion experiments with endogenous proteins, which were designed by RD. RB performed electron microscopy. MV and IK interpreted the electron microscopy experiments. RD wrote the materials and methods section and submitted the sequences to Genbank. JM v/d L composed the peroxicretion protein test set. MA performed MS/MS experiments. All authors contributed to editing and writing of the paper.
